# Supplementary material for: Macrophage-Derived Exosomal MALAT1 Induced by Hyperglycemia Regulates Vascular Calcification Through miR-143-3p/MGP Axis in Cultured Vascular Smooth Muscle Cells and Diabetic Rat Carotid Artery
Source: Cells. 2025 Dec 15;14(24):1995. doi: 10.3390/cells14241995 (PMC12731768; doi:10.3390/cells14241995)
Supplement: Supplementary file 1 [file cells-14-01995-s001.zip › cells-3966062-supplementary.pdf]

Histological and immunofluorescence staining

The tissue samples of carotid artery were harvested and treated overnight in 4% paraformaldehyde and then embedded in paraffin. The tissues were cut into 5-µm-thick sections and incubated with the primary antibody, MGP polyclonal antibody (Invitrogen, Thermo Fisher Scientific, Waltham, MA, USA) or monoclonal anti-ß-actin antibody (Sigma-Aldrich, St. Louis, MO, USA) at 4℃ for 12 hours. The sections were then washed thrice with PBS, then incubated with fluorescence-conjugated secondary antibody in PBS for 1-2 hours at room temperature in the dark and then stained with DAPI to visualize the nuclei. The sections were mounted with a coverslip and examined under a fluorescence microscope. Images were taken from at least three random fields for each sample.

Alizarin red staining of carotid artery

The staining solution was prepared by dissolving 2 g of Alizarin Red S powder (Sigma-Aldrich, Cat. No. A5533) in 100 mL of distilled water to create a 2% (w/v) solution. The solution was mixed thoroughly on a magnetic stirrer. The pH of the solution is critical for staining specificity and was carefully adjusted to 4.2 using 0.1% ammonium hydroxide (NH_4OH). The final solution was passed through a 0.45 µm syringe filter to remove any particulate matter and was stored at room temperature in the dark. Formalin-fixed, paraffin-embedded rat carotid arteries were sectioned at a

thickness of 5 µm using a microtome and mounted on positively charged glass slides. The slides were then incubated overnight at 60℃ to ensure tissue adherence. Prior to staining, the sections were deparaffinized and rehydrated through a series of graded solvent washes as follows: Xylene: 2 changes, 10 minutes each; 100% Ethanol: 2 changes, 5 minutes each; 95% Ethanol: 1 change, 3 minutes; 80% Ethanol: 1 change, 3 minutes; 70% Ethanol: 1 change, 3 minutes; Distilled water: 2 changes, 5 minutes each. Following rehydration, the slides were fully immersed in the prepared 2% Alizarin Red S solution (pH 4.2) and incubated at room temperature for 3-5 minutes. The staining progress was monitored intermittently under a light microscope to prevent overstaining. Once the calcium deposits appeared as a vivid orange-red precipitate, the slides were immediately removed from the staining solution and rinsed gently but thoroughly in several changes of distilled water to remove excess, unbound dye. After rinsing, the sections were rapidly dehydrated to preserve the stain. Slides were briefly blotted to remove excess water and then processed as follows: Acetone: 1 change, 30 seconds; Acetone/Xylene (1:1 v/v): 1 change, 30 seconds; Xylene: 2 changes, 3 minutes each. Finally, the slides were coverslipped using a synthetic, resinous mounting medium (e.g., DPX). The slides were allowed to dry completely in a fume hood before imaging.

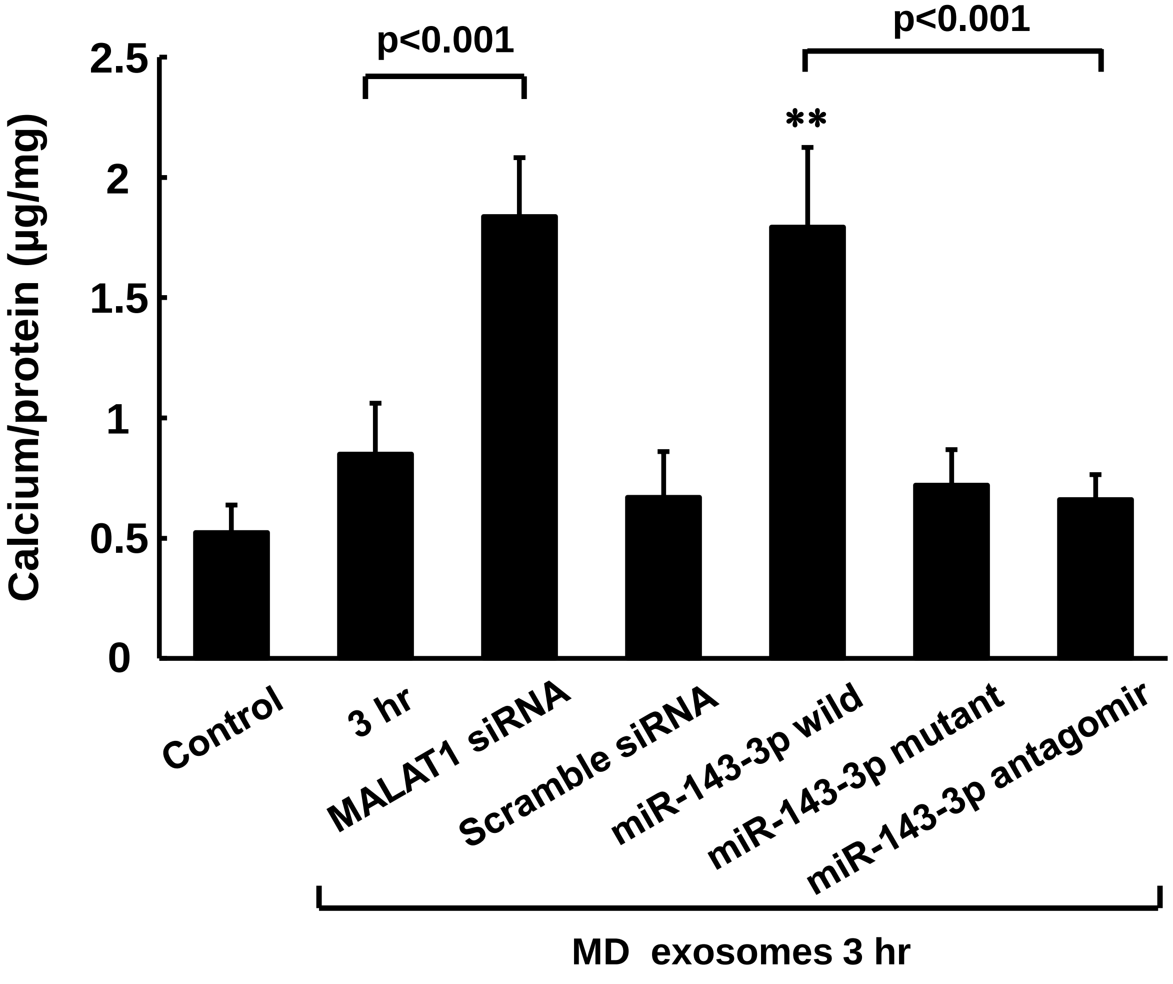

Supplementary Figure S1

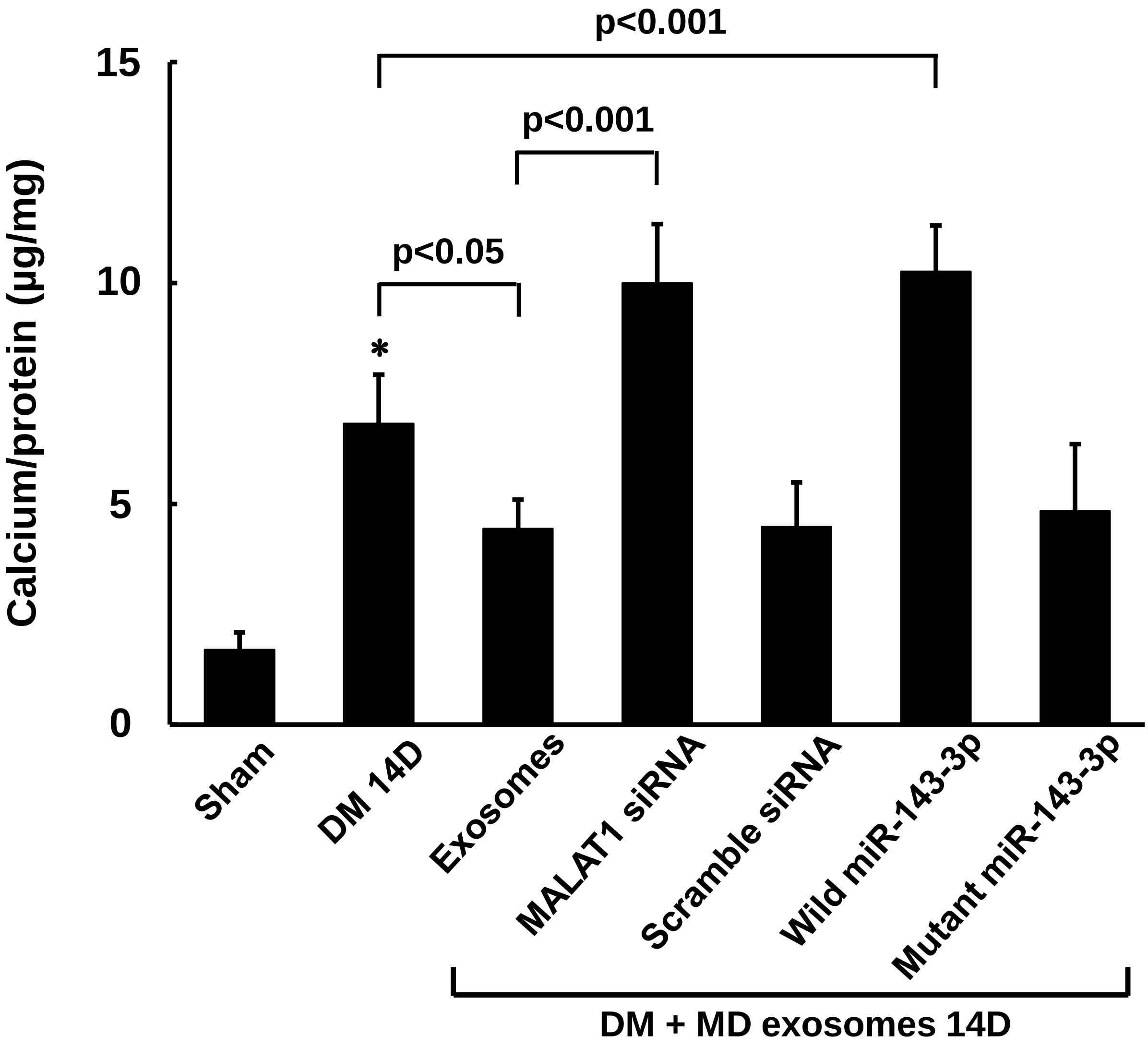

Supplementary Figure S2

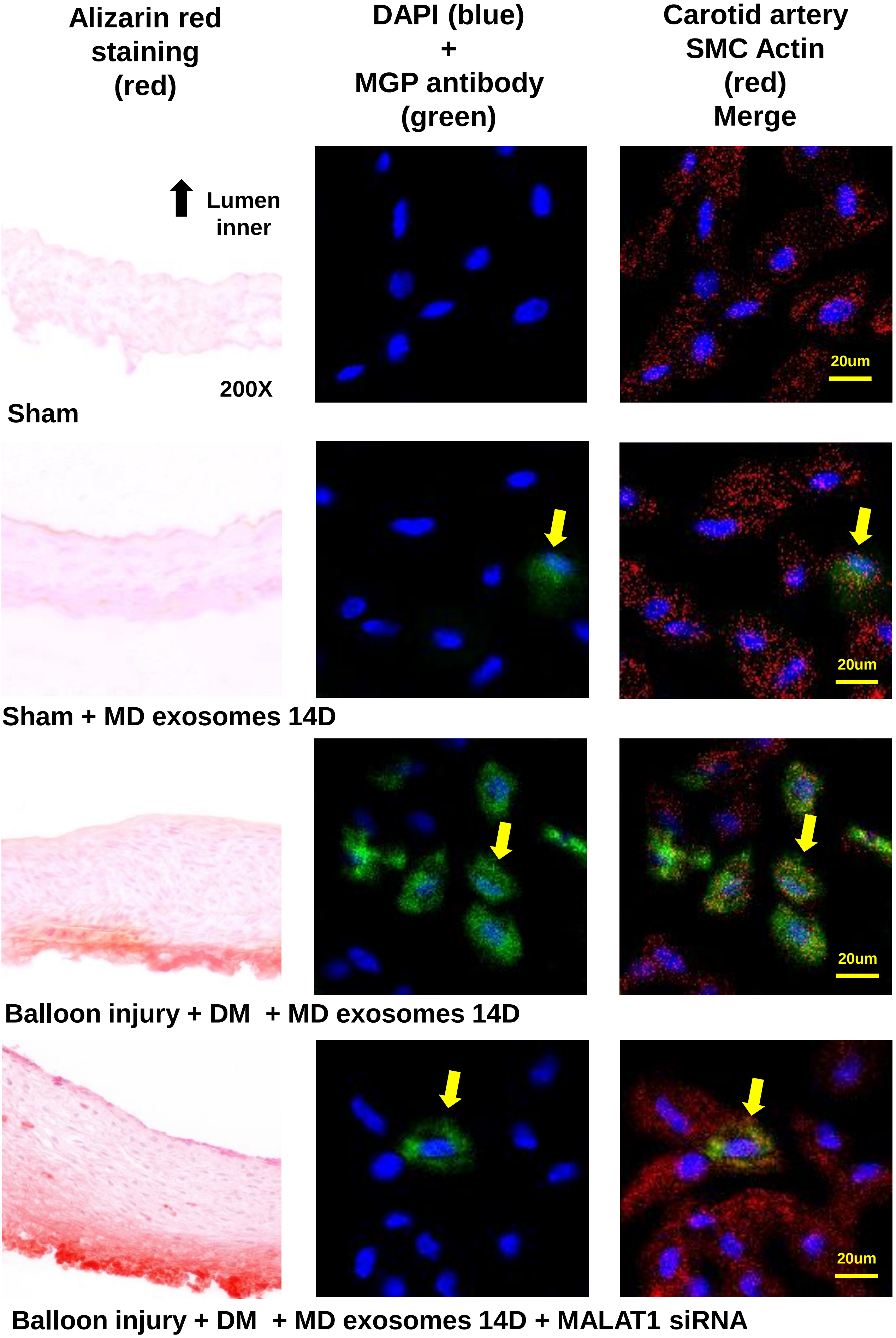

Supplementary Figure S3

Supplemental Figure Legends

Supplementary Figure S1: Effect of macrophage-derived (MD) exosomes on the calcium content of cultured vascular smooth cells. Fifty μg macrophage-derived exosomes extracted from macrophages under 25 mM glucose treatment for 3 h was used. Calcium content was measure by calcium colorimetric assay. ** P<0.001 vs 3 hr. N=3 per group.

Supplementary Figure S2: Effect of macrophage-derived (MD) exosomes on the calcium content of carotid arteries in diabetic rats after balloon injury of carotid artery for 14 days. *P<0.001 vs sham. N=5 per group.

Supplementary Figure S3: The macrophage-derived exosome increases MGP protein labeling after balloon injury of the carotid artery for 14 days. Immunofluorescence staining of the intimal area was performed at 14 days after balloon injury of the carotid artery. Labeling of MGP increased after balloon injury and macrophage-derived exosomes therapy. Silencing of MALAT1 by MALAT1 siRNA decreased the labeling induced by balloon injury of the carotid artery. The MGP positive labeling cells were vascular smooth muscle cells, which were proved by positive anti-SMC actin staining. Alizarin red staining showed increase of calcium content signaling in the carotid artery after balloon injury in diabetic rats treated with macrophage-derived exosomes for 14 days and MALAT1 siRNA enhanced more calcium content in the carotid artery.
